# Building a culture of healing to support nurse faculty and staff well‐being in the aftermath of COVID‐19

**DOI:** 10.1002/nop2.1584

**Published:** 2023-01-03

**Authors:** Laura Sinko, Beth Heuer, Lisa Johnson, Jennifer Brown, Kaitlyn Heron, Marjorie Lehigh, Susan Gresko, Susan Dickey, Brenda Teichman, Krista Schroeder

**Affiliations:** ^1^ Department of Nursing Temple University College of Public Health Philadelphia Pennsylvania USA

The COVID‐19 pandemic has greatly impacted individuals at all levels of higher education, leading to calls for universities to prepare for the long‐term impacts of the pandemic on student, faculty, and staff well‐being (Knight et al., 2021). While the majority of COVID‐19 research relating to higher education has been devoted to understanding its impact on student and institutional outcomes, little attention has been paid to how the pandemic has impacted faculty well‐being and how this may trickle down to impact student learning and university climate.

We are a group of nursing faculty from a public university, who have witnessed the traumatic impact that the pandemic has had on our department and others across the nation. We recognize that to provide the highest quality experience for nursing students in the uncertain aftermath of the pandemic, we need to address the harm we have experienced in our faculty and staff roles, rebuild community, and think of creative solutions to develop and model a culture of healing. The purpose of this editorial is to share observations and perspectives on the experiences of nursing faculty during the pandemic, and to propose potential efforts to support healing and continued educational excellence moving forward. Our proposals are guided by a combination of evidence, clinical expertise, and personal experiences across higher education.

## 1 THE UNIQUE IMPACT OF THE COVID‐19 PANDEMIC ON NURSING FACULTY AND STAFF

While many in higher education were able to transition solely to online learning in the height of the COVID‐19 pandemic, those in healthcare professions were unable to do so. Nursing education hinges on practical, in‐person clinical learning to meet licensing needs, many of which are embedded in environments at highest risk for pandemic‐related trauma exposure. As a result, for many nursing faculty, numerous unknowns existed at the onset of the pandemic. Many needed to quickly modify curricula delivery, balance student and personal safety with degree requirements, leverage new partnerships to provide clinical experiences, and learn how to address the needs of students thrust into a traumatic new learning environment. In addition, rapidly changing and unclear safety guidelines contributed to institutional betrayal and chaos across academic communities, with many looking to healthcare colleagues for support and consultation. Additional demands outside of work, such as caregiving‐related demands or the pressure to serve as a leader in one's community, blurred the lines between work and home life, leading to burnout and increased exposure to harm and trauma.

## 2 THE RISK OF NOT ADDRESSING FACULTY WELL‐BEING CONCERNS

**FIGURE 1:**
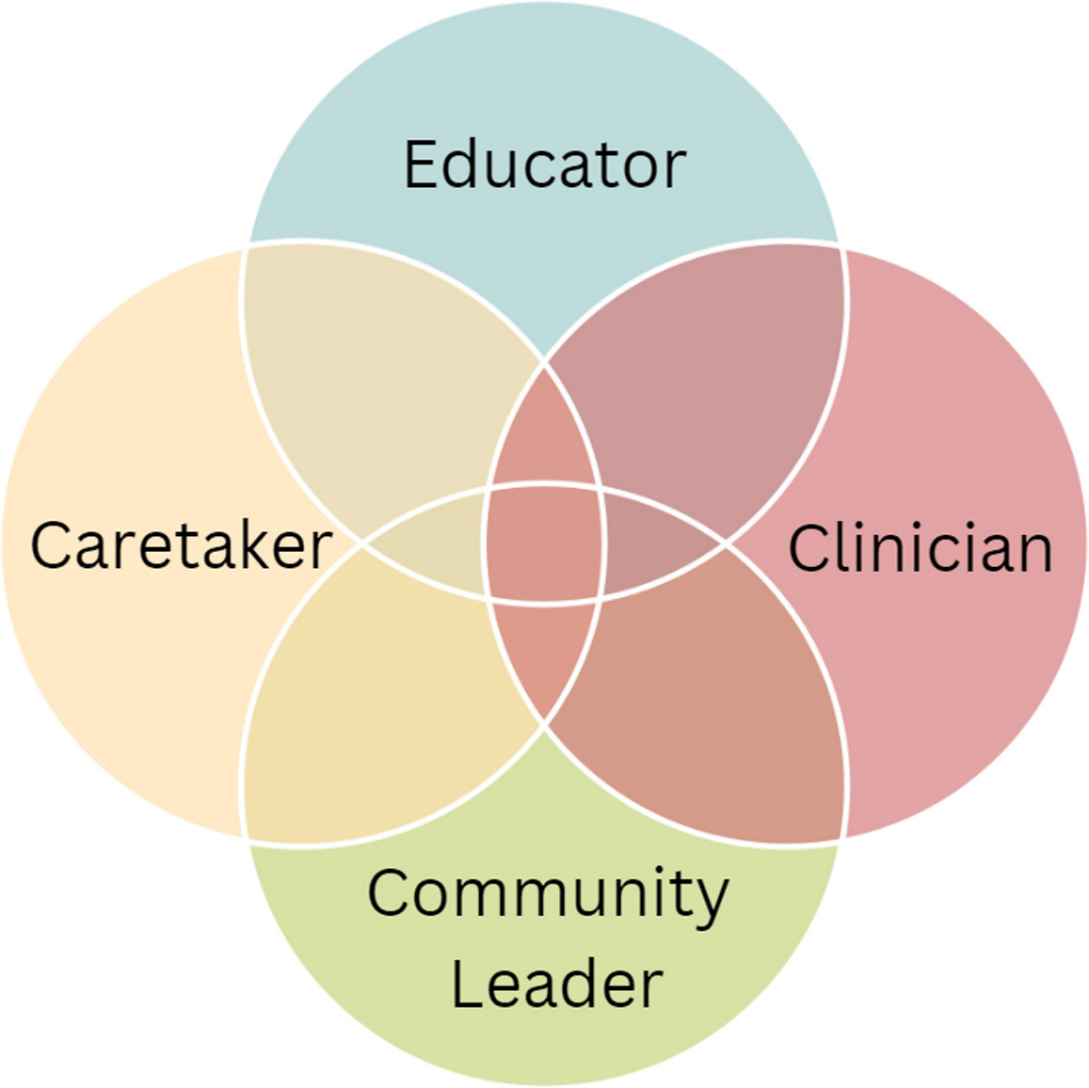
Nurse faculty's varied roles during the pandemic

The impact of nurse faculty's roles during the pandemic (see Figure 1) and competing demands across those roles has created a lasting impact on faculty and staff well‐being. For many, new onset or worsening mental health symptoms were experienced as a result, including anxiety, recurring dreams, ruminating thoughts, and feelings of anger, guilt, loss, sorrow and grief.

Even prior to the pandemic, the profession engaged in discussions around burnout, retention, and satisfaction within the nursing faculty role. Hence, to support the future of nursing education, nursing schools and departments need to acknowledge the trauma and harm faculty and staff have experienced during COVID‐19 and work towards a culture of healing.

## 3 POTENTIAL SOLUTIONS

Below, we outline potential solutions that may support healthy post‐pandemic academic nursing climates and center the healing needs of nursing students, faculty, and staff.

### 3.1 Employ trauma‐informed care principles to prevent retraumatization

Understanding and introducing trauma‐informed care principles at the organizatioal level is one potential way to rebuild trust and strengthen communication in academic nursing settings. Expanding on the approach of Goddard et al. (2021) in response to students' COVID‐19 trauma, universities must realize COVID‐19 trauma and its impact, recognize signs and symptoms of trauma in faculty and staff, respond by integrating knowledge on trauma‐informed practices, and resist retraumatization.

A trauma‐informed nursing department employs teaching strategies which strive to mitigate power dynamics, provide transparency, voice, and choice when possible, and sensitively addresses historical harms. They provide clear and consistent expectations, share resources, and offer clear channels for faculty and staff feedback to consistently allow for responsiveness, approach improvement, and proactive responses to challenges that arise. Departments of nursing can also take a trauma‐informed approach by actively working to create a culture that prevents and reduces harm by fostering spaces inclusive of all identities, being actively accountable for actions that perpetuate harm, and building community before harm occurs.

### 3.2 Implement community building principles and mechanisms to address harm

While it is tempting to want to leave the trauma perpetuated by COVID‐19 in the past, before we move forward, we must first create formal channels to acknowledge and address the harm experienced at all levels. One way we might do this is employing community building and restorative justice‐inspired infrastructure to facilitate critical dialogues, center harm, and rebuild community (Karp, 2019). Studies show that utilizing community‐building and debriefing practices in academic environments positively affect cognitive and skills development, while also creating space to discuss psychological, emotional, and moral responses to one's lived experience (Bender & Walker, 2013). Addressing harm in this way can allow those who choose to participate to feel heard and contribute to solutions to improve department or school climate. Indeed, if we are looking to improve academic culture and proactively respond to personal and professional challenges caused by COVID‐19, we need to be intentional.

Community‐building circles can help mitigate power dynamics, cultivate relationships across levels, and create a culture of prevention and healing (Karp, 2019). In the end, by facilitating these circles across department or school levels regularly, universities can show that they care about academic community member well‐being while also partnering to create interventions going forward. If the harm they experienced is taken seriously, faculty and staff will be more likely to feel respected, safe, and supported‐values that will be replicated in their relationships with students and each other. This will help build a new foundation to meaningfully grow from, while also creating a shared language to proactively address future harm.

### 3.3 Promote self‐ and community care, but recognize institutional support is critical

When faculty draw on the American Nurses Association (ANA, 2015) *Code of Ethics for Nurses* as a non‐negotiable professional standard of care, they can find inspiration that they are part of a profession bigger than themselves as individuals. Provision 5 requires that “The nurse owes the same duty to self as others including the responsibility to promote health & safety, preserve wholeness of character & integrity, maintain competence, and continue personal & professional growth” (ANA, 2015, p. 19). Such guidance provides a reliable source, to make room for care and growth during challenging situations.

Nursing faculty must embrace principles of self‐ and community care and model these imperatives to our students. One element of this involves critical self‐reflection, which can help faculty gain insight into current practices, identify needed supports, recognize how they can support other faculty members, and find new purpose in their work. In addition, workplaces should consider employing psychological first aid, a response designed to help pandemic workers and survivors with safety and stabilization and to connect individuals to helpful mental health resources. Examples of these efforts include hosting facilitated group discussions and promoting activities that enhance self‐efficacy and community resilience. Finally, leadership should consider enhancing the ability for job crafting, where possible, to reflect changing circumstances and needs. Job crafting involves personalized, proactive redesign of one's job to enhance autonomy and job satisfaction. Adoption of job crafting opportunities for faculty is a viable structural change in the university system that can help promote more opportunities for balance, meaningful engagement, and self‐care.

Individual‐level actions are most effective when occurring in a robust culture that supports the flourishing and dynamic needs of its members. As a result, universities must also critically examine their role in perpetuating trauma and supporting community well‐being. Such an examination must be deep and critical ‐ as superficial or performative efforts will only increase distress and disengagement. While easy small changes can be helpful in fostering a positive environment, deeper structural changes must also be considered to truly shift to a healing culture. A school or department must consider how its policies, culture, expectations, norms, and workload affect faculty wellness and burnout. When opportunities for improvement are illuminated, change should be supported by leadership and pursued in a faculty‐driven and evidence‐based manner that builds from the robust literature on trauma‐informed organizations.

## 4 HEALING IS A JOURNEY, IT DOES NOT HAPPEN OVERNIGHT

The COVID‐19 pandemic has created a long‐lasting impact on nursing education, affecting students and faculty alike. As a profession, we cannot rush to “return to normal” without taking the time to learn and reflect. COVID‐19 has been a major disruptor that poses opportunities for innovation and a recentring. Nurses have placed their lives on the line and sacrificed their work‐life balance and self‐care practices throughout the pandemic, creating a worsening mental health crisis in the profession. An enhanced emphasis on well‐being in nursing is needed, starting with re‐educating ourselves on what well‐being looks like for us and passing that knowledge along to our students. For us to be a healing force for the world, we must first look inward and heal ourselves‐‐but we do not have to do it all on our own. We can be guided by rich evidence about trauma and recovery and look to health care and institutional leaders to work towards a culture of healing for all.

## AUTHOR CONTRIBUTIONS

LS conceptualized the editorial. All authors contributed to writing the original draft and reviewing and editing the draft. BH, KS and LS refined the manuscript based on all authors' feedback. All authors approved the final manuscript.

## CONFLICT OF INTEREST

We have no conflicts of interest to disclose.

## ETHICAL APPROVAL

No ethical approval was required for this editorial.

## Data Availability

No data is associated with this editorial.

## References

[nop21584-bib-0001] American Nurses Association . (2015). Code of ethics for nurses with interpretive statements. American Nurses Publishing Code of ethics for nurses with interpretive statements (view only for members and non‐members) (nursingworld.org).

[nop21584-bib-0002] Bender, A. , & Walker, P. (2013). The obligation of debriefing in global health education. Medical Teacher, 35(3), e1027–e1034. 23102161 10.3109/0142159X.2012.733449

[nop21584-bib-0003] Goddard, A. , Jones, R. W. , Esposito, D. , & Janicek, E. (2021). Trauma informed education in nursing: A call for action. Nurse Education Today, 101, 104880. 33798984 10.1016/j.nedt.2021.104880

[nop21584-bib-0004] Karp, D. R. (2019). The little book of restorative justice for colleges and universities: Repairing harm and rebuilding trust in response to student misconduct. Simon and Schuster.

[nop21584-bib-0005] Knight, H. , Carlisle, S. , O'Connor, M. , Briggs, L. , Fothergill, L. , Al‐Oraibi, A. , Yildirim, M. , Morling, J. R. , Corner, J. , Ball, J. , Denning, C. , Vedhara, K. , & Blake, H. (2021). Impacts of the COVID‐19 pandemic and self‐isolation on students and staff in higher education: A qualitative study. International Journal of Environmental Research and Public Health, 18(20), 10675. 34682418 10.3390/ijerph182010675 PMC8535702

